# The Provision and Utilization of Traditional Korean Medicine in South Korea: Implications on Integration of Traditional Medicine in a Developed Country

**DOI:** 10.3390/healthcare9101379

**Published:** 2021-10-15

**Authors:** Jieun Park, Eunhee Yi, Junhyeok Yi

**Affiliations:** Research Center for Traditional Korean Medicine Policy, Korea Institute of Oriental Medicine, Daejeon 34054, Korea; ehyi@kiom.re.kr (E.Y.); hanidoc@kiom.re.kr (J.Y.)

**Keywords:** Traditional Korean medicine, health care utilization, monitoring, national health insurance, Republic of Korea

## Abstract

Traditional Korean medicine (TKM) is formally integrated into the Korean national health system and monitored through the systematic and computerized system, which could grasp the whole medical services utilization in Korea. This study analyzed TKM resources as input and utilization as output using data from 2008–2017 and compared them to Conventional Medicine (CM). As a result, 25.4% of Koreans utilized TKM yearly, and the proportion of TKM medical expenditure (ME) to total ME in national health insurance was around 4% between 2008 to 2017. The proportion of ME has been stagnating or decreasing over the past ten years. Primary users are the elderly, women, and patients with musculoskeletal diseases. The Korean Ministry of health and welfare has also developed and operated programs that have taken advantage of the strengths of TKM. This study analyzes the current status of TKM in Korea comparing with that of CM. It also explores how and why the patterns of TKM and CM are different. Although the study focuses on input and output indicators, it also highlights the challenge of evaluating whether these lead to outcomes. Lastly, it seeks to inform relevant authorities of the importance of monitoring roles and evidence-informed policymaking.

## 1. Introduction

Many Asian countries are integrating traditional medicine (TM) into their health systems [1,2]. To achieve this goal, it is essential to grasp the current situation reasonably. It can help policymakers design policies that reflect reality better based on more objective evidence. For this reason, many Asian countries strive to establish infrastructure and develop a system to monitor the current status of TM in their health system [3,4]. However, few countries properly grasp the snapshots of TM, which works as a reason of why many Asian countries are having difficulty designing and implementing appropriate TM policies [5,6].

Republic of Korea (Korea) is one of the countries in which TM is formally integrated into the national health system (NHS) and has well-established national statistics. In addition, Korea operates a dual system in which CM (Conventional medicine) and TKM (Traditional Korean Medicine) services are provided separately within one NHS. In other words, TKM doctors provide medical services such as acupuncture, moxibustion, cupping, chuna, or herbal medicines (HM) under governmental regulation. While CM doctors do not provide such services, they diagnose with modern medical devices and prescribe medicines except for HM. 

The characteristics of these different health care provisional systems also have different effects on medical utilization. Therefore, designing sophisticated policies tailored to CM and TKM characteristics is one of the objectives of policymakers. Consequently, it is common in Korea to monitor the current status of CM and TKM separately, and this monitoring system makes it possible to compare CM and TKM directly. Nevertheless, few researchers have analyzed CM and TM together on the same line, and the status of TM is not well known [7,8,9].

The input and output of healthcare are proxy indicators representing the preference of the public and effectiveness of the services as well as policy results. For this reason, many countries regularly monitor the health care status and compare it with other countries. [10]. In particular, countries considering integrating TM into the NHS, as is the case of Korea, can give various implications in establishing their monitoring healthcare system and prioritizing policies.

The purpose of this study is to analyze the health care resources as input and the health service utilization as output using national statistics. In addition, these results will be compared to that of CM, and be explored as to how and why both patterns are different. Lastly, the roles and challenges of TKM in the health system in Korea will be discussed.

## 2. Materials and Methods

As data were distributed in several places, the researcher collected each reference and extracted the data necessary for the analysis. Principal data sources were the Korean Traditional Medicine Yearbook [11], the National Health Insurance Statistical Yearbook [12], the annual statistics on health and welfare [13], the statistics on medical expenditure [14], the statistics on prescribed medicines [15], and the survey on patients [16], which are publicly available.

Extracted data were classified into health care resources as input, and health service utilization as output. Input indicators include the number of health professionals and facilities as health resources, and output indicators include medical expenditure (ME), prescription of herbal medicines, and disease characteristics as health service utilization in CM and TKM. Basically, CM and TKM status are compared. Since Korea has several types of health insurance, extracted data were reconstructed by health insurance. In the case of ME, utilization pattern by age was analyzed additionally.

The value in 2008 and 2017 was extracted, and the annual average growth rate was calculated to compare their changes over time. The chi-square test was performed in the case of sample data [16]. No statistical analysis was performed as the rest of the references were data reflecting the total subjects.

## 3. Results

### 3.1. Human Resources and Facilities in TKM

Table 1 summarizes the current status and changes in human resources and facilities in TKM over the past decade in Korea. In terms of human resources, based on the number of licensed TKM personnel in Korea, TKM doctors accounted for 5.5% among the total health personnel, including nurses (51.2%), CM doctors (27.8%), CM + HM pharmacists (8.4%), and dentists (7.1%), in 2017. The number of licensed TKM doctors increased by 9809 over the past ten years, from 14,818 in 2008 to 24,627 in 2017. 

If the analysis is limited to the doctors (CM, TKM, and dental medicine), TKM doctors totaled 16.4% in 2017, which meant an increase of 3.0% *p* from 2008, 13.4%. The number of doctors who hold licenses for both CM and TKM increased from 189 in 2008 to 316 in 2017. If we analyze data including only pharmacists (CM and HM), the number of licensed HM pharmacists was 2404, increasing 1.96 times in 2017 compared to 2008. 

Regarding health facilities, the proportion of TKM facilities was higher than that of TKM doctors for ten years (2008–2017). While TKM facilities have increased, on the whole, the proportion of total TKM facilities has remained in the early 20% level for the past ten years (Table 1). TKM clinics consist of the most significant number (94.5%) of TKM facilities; over 300 TKM hospitals specialize in TKM treatment and have increased to over 300 for the first time in 2017. The annual average growth rate, or Compound Annual Growth Rate (CAGR), also shows a different pattern by the type of facilities over the past decade. The total number of health facilities in Korea has increased by 2.1% between 2008 and 2017 (data not shown). Among these facilities, TKM clinics showed a 2.5% growth rate of the CAGR compared to the CM clinics (1.7%, data not shown). TKM hospitals increased 8.8% from 2008 to 2017, which shows the highest growth rate among health facilities in Korea.

### 3.2. Medical and Pharmaceutical Expenditure in TKM

Under the universal health coverage system, most of the residents in Korea can access medical services regardless of the type of health insurance. As a result, more than 90% of the whole population visit health facilities more than once a year [12]. Limited to TKM in NHI, about 12.9 million people (25.4%) visited TKM doctors in 2017 [12]. As age increases, TKM is utilized more frequently. From a gender perspective, the tendency for women to utilize more medical services than men was similar in both TKM and CM, but the proportion of women utilizing TKM (64.5%) was higher than CM (56.8%) (*p* < 0.001).

The ME in TKM was compared according to the type of health insurance. The ME, which is the sum of the payment by insurers and out-of-pocket payment by users, incurred by each health insurance, is presented in Table 2. The total amount of TKM ME in 2017 was USD 2.24 billion, which is equivalent to 3.6% of the total ME, including National Health Insurance (NHI), which accounted for 77.6% among total TKM ME, Medical Aid (5.1%), Workers’ Compensation (0.1%), and car insurance (17.2%).

Several factors could explain the reason for the annual increase in ME. The primary reasons are the increase in medical utilization due to the increase in the elderly population and the expansion of insurance coverage. However, each situation varies depending on the type of insurance. Focusing on NHI, the CAGR of TKM ME (2008–2017) seems to have increased to 7.2% and is lower than CM (8.0%). In addition, the proportion of TKM ME in the NHI decreased every year since 2013 (4.2%) to 3.6% in 2017 (data not shown).

On the contrary, in the other types of health insurance, the share of TKM was much less and CM was the same, but CAGR was higher than CM. In the case of Medical Aid, which is public health insurance to ensure access to health care for low-income groups, TKM ME accounted for 2.3% in Medical Aid in 2017. In terms of the rate of ME growth, CAGR of ME in TKM was 8.2% (2008–2017), which is 2.9% *p* higher than CM in the same type of insurance. In addition, for car insurance, the CAGR for TKM ME was the highest among the total type of insurance schemes. In 2017, the TKM ME in car insurance amounted to USD 495.7 million, consisting of 31.8% of the total ME in the car insurance showing 26.3% *p* higher than CM, and is equivalent to 22.2% TKM ME in NHI. 

In terms of HM, the types of HM covered by NHI are limited to 67 single herbal extracts and 56 prescriptions (mixtures of single herbal extracts) in Korea [17]. As a result, many HM have been prescribed decoctions or other HM that NHI does not cover. Therefore, there is a limit to understanding the total prescription of HM. Focusing on covered HM, a total of USD 31.7 million were spent in 2017, accounting for 0.21% of the total pharmaceutical expenditure (PE), which is much less than 3.6% of the share of TKM ME in NHI (Supplementary Table S1). In other words, it indicates that most of the TKM ME is spent on medical interventions, such as acupuncture, moxibustion, and cupping, not HM.

**Figure 1:**
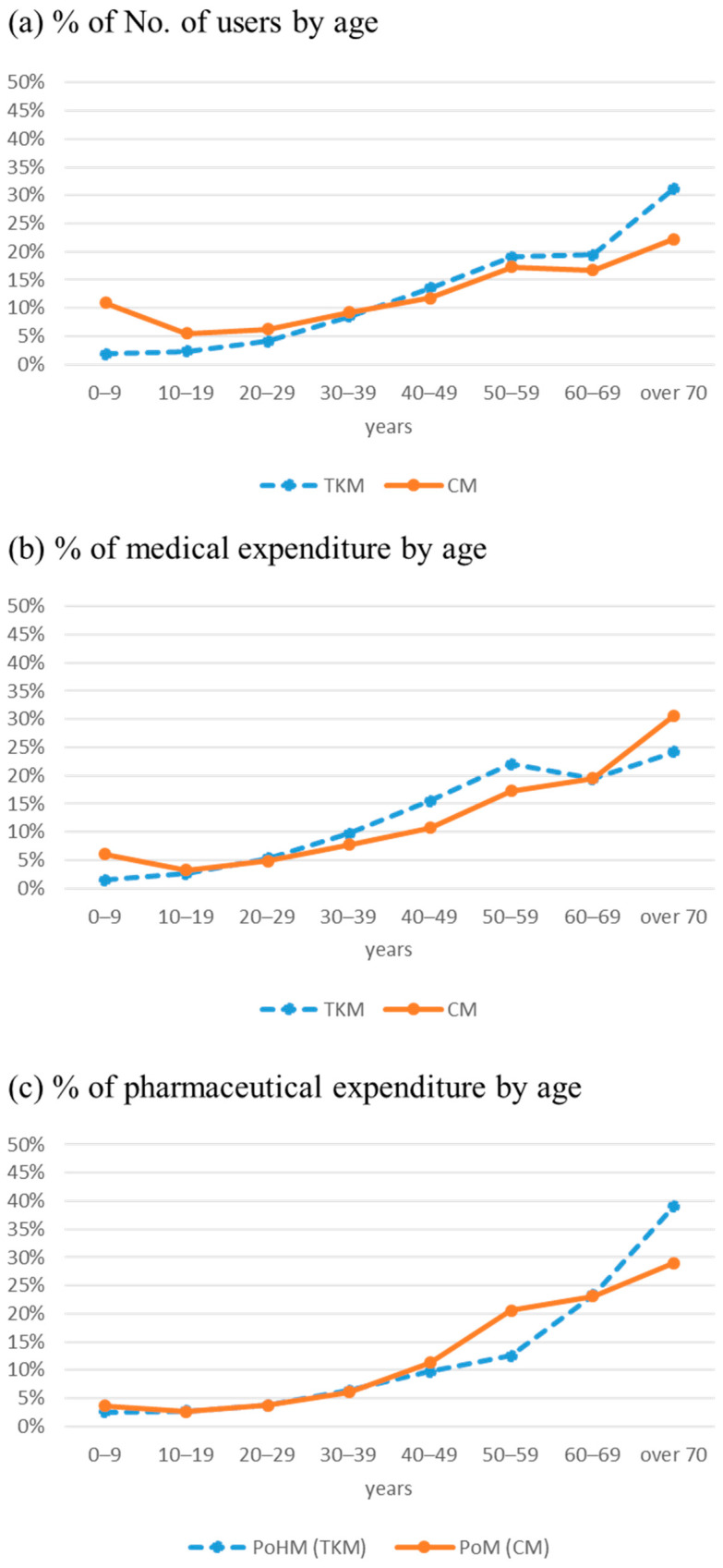
% of the number of users ((**a**), **top**), % of medical expenditure ((**b**), **middle**), and % of pharmaceutical expenditure ((**c**), **bottom**) in Conventional medicine (solid line) and Traditional Korean Medicine (dotted line) by age in the National health insurance in 2017. Abbreviations: CM, Conventional medicine; TKM, Traditional Korean Medicine; PoM, Prescription only Medicines; PoHM, Prescription only Herbal Medicines.

The distribution of the number of users and the amount of ME and PE in percent by age are shown in Figure 1. The proportion of users (a) and ME (b) by age in CM show a typical J-curve (straight line). It describes that the utilization of medical services for children under 10 is higher than those for teenagers and increases as age increases. However, the pattern in TKM (dotted line) is different from the overall ME in CM. The proportion of users is the smallest under ten and increases linearly with age. TKM ME is the lowest under ten years old, peaking in their fifties and decreasing in their sixties. In summary, TKM is higher than CM in the proportion of users over 65 years old (23.1% (CM) vs. 41.8% (TKM), *p* < 0.001); however, CM is higher than TKM in the proportion of ME (40% (CM) vs. 33.6% (TKM)).

Regarding PE, it tends to increase with age, and the patterns of CM and TKM were similar (Figure 1c). However, HM was prescribed at a higher rate in the elderly over the age of 60 than prescription-only medicines (PoM) that are prescribed in CM. When limited to the elderly, 52% of the total PE for CM and 62% of the total expenditure of HM for TKM were spent at the age of 60 and over, respectively. In other words, those in their 60s and younger visiting CM facilities account for 48% of total PE. In contrast, those in their 60s and younger visiting TKM facilities account for 38% of the total expenditure of HM.

### 3.3. Utilization of TKM by the Type of Diseases 29

Most TKM users visit TKM doctors in Korea due to musculoskeletal-related disorders (MRD), such as back pain and arthritis, closely related to geriatric diseases. A total of nine MRD are ranked among the top 10 frequent diseases in TKM utilization. In addition, approximately 77.2% of the total TKM ME in the NHI was spent on MRD. In other words, the remaining 22.8% of TKM ME in NHI account for TKM utilization of non-MRD in Korea. On the contrary, only 15% of the ME comprises MRD in CM in NHI.

Besides MRD, the outpatients mostly visited TKM doctors due to dyspepsia, common cold, abdominal and pelvic pain, headache, and allergic rhinitis (health complaints ordered by incidence). On the other hand, the top 5 reasons for visiting CM clinics were associated with bronchitis, rhinitis, respiratory infections, or periodontal disease. 

In the case of hospitalization, the top five reasons for TKM except for MRD were hemiplegia, cerebral infarction, dementia, cerebrovascular disease sequelae, and facial nerve disorders (health complaints ordered by incidence). On the contrary, patients admitted to hospitals suffered from infectious gastroenteritis and colitis, cataract, pneumonia, hemorrhoids, and dementia (health complaints ordered by incidence) (Supplementary Table S2).

## 4. Discussion 

This study analyzed and compared the input (resources) and the output (utilization) of TKM services with CM in Korea. Although the share of TKM resources among total medical resources has increased, the share of TKM ME did not increase accordingly and even decreased in NHI, which consists of almost 90% of NHS.

In summary, one in four Koreans utilized TKM yearly with higher proportions in the elderly, women, and patients with MRD than that of CM. TKM doctors accounted for 16.4% of total doctors and 21.8% of total health care facilities, considering the sum of both TKM hospitals (8.7%) and clinics (22.6%), respectively. The share of TKM ME ranged from 0.6% to 31.8% as a function of the insurance.

Focusing on NHI, the largest health insurance type in Korea, TKM ME by age did not show a general J-curve shape. This could be explained by several factors. First, in terms of demographic structure, there are fewer than 2.9 million people in their 60s than in their 50s in Korea. In addition, the actual number of total medical service users in their 60s is also lower than in their 50s. The ME of CM in their 60s was only slightly higher than those in their 50s. The 60s population might also spend more money on their medical services due to severe diseases such as cancer or cerebrovascular diseases requiring intensive and high-tech medical services, which are limited in TKM treatment. 

Second, it might reflect that the disease characteristics of patients utilizing TKM are different from CM. As mentioned above, the main reason for the utilization of TKM is MRD. Therefore, the shape of ME by age in TKM is similar to that seen in the MRD ME model, which mainly includes patients with back pain [18]. Likewise, it is quite similar to the age-specific ME in car insurance, which consisted of a high proportion of patients with MRD (data not shown). It is well known that the main reasons for using traditional and complementary medicine are MRD and pain in many countries [19,20,21]. This may raise questions about whether TKM is primarily effective for MRD or why it is generally not being used for other diseases except for MRD. One plausible explanation is that the level of coverage in terms of the type of services and fees might impact medical services utilization. For example, TKM services in NHS mainly cover acupuncture, moxibustion, cupping, and chuna, which are more advantageous in treating MRD. In addition, TKM doctors are not legally authorized to prescribe medicines used in CM such as antihypertensives, or order simple blood tests, X-rays, and expensive MRI and CT, which again act as obstacles in expanding and diversifying TKM services. Furthermore, these differing objectives for visiting health facilities and types of provisional services between TKM and CM might reduce the possibility of cooperation by decreasing the common denominator. In terms of HM, a limited number of HM [17] are covered by NHI while the rest, including decoction, are not. Therefore, patients other than MRD who might benefit from HM are sometimes reluctant to take HM due to lack of coverage. In contrast, car insurance is an example that shows that the level of benefit coverage differs in the patients’ medical service utilization pattern. Unlike other types of health insurance, car insurance covers several more TKM interventions and HM, including decoction. As a result, we can see that the rate of utilizing TKM in car insurance is much higher (31.8%) than that of other types of insurance (0.6~3.6%) (Table 2).

There are a variety of perspectives in interpreting the current statistics. Although TKM plays gatekeeping in MRD, it does not appear to be in non-MRD. Patients with non-MRD might visit TKM facilities as they are not satisfied with treatment outcomes in CM rather than preferentially utilizing TKM [22]. Under this logic, TKM might function as to complement, not to substitute [23], although TKM has independent medical authority as medical professionals in Korea. Moreover, the TKM and HM industries have been replaced with health supplements and home medical equipment industries [24,25]. This situation can adversely affect the future position of TKM. 

Another point to note is that the younger generation is decreasingly not utilizing TKM compared to the older generation [22]. This also foreshadows a grim outlook as TKM seems to have failed to garner potential users’ interest. It can lead to less active public support for expanding TKM coverage policies only because it is traditionally transmitted therapy. 

Countries sometimes intervene politically to maintain the quality and supply of TM above a certain level. A typical example is the establishment of TM department in National universities and public hospitals. However, there is only one National university with TKM curriculum in Korea (ten National universities with CM curriculum) [26], and only 19 (14.7%) out of 129 public hospitals provide TKM services as of 2019 [27]. This situation shows a limit to expanding the role of TM led by the state in the Korean medical market, where CM and the private sector dominate. Eventually, this situation acts as one of the reasons for the decrease in public access to TKM.

Nevertheless, TKM still has strengths in the field of the elderly and MRD, as the elderly are familiar with TKM, making it possible to take these advantages in promoting health programs. Korea currently has operated more than 200 TKM community-based programs for citizens [28,29]. The Korean government also began to implement a pilot program for home-visit TKM services for people with mobility difficulties in 2021 [30]. TKM doctors can directly provide acupuncture, cupping, and moxibustion during visits, unlike CM doctors, who are limited in the medical treatment they can provide at patients’ homes. 

The Korean government has also invested annually about USD 85 million in public research funds to find and establish scientific evidence in TKM [11]. One of the representative achievements is the development of clinical practice guidelines in 30 diseases that TKM has strength in [31]. It aims to make evidence-based TKM a reality, and the development of a new payment model is being investigated based on clinical practice guidelines [32].

Lastly, in terms of statistical monitoring, TKM has a long way to go when evaluating outcomes such as effectiveness, safety, person-centered care, efficacy, and time [33]. As is well known, input and output of health care are relatively easy to measure; however, measurement of outcomes is more complicated and controversial, and the same is true of TKM. The majority of TKM users concurrently utilize CM and TKM, which makes it challenging to select outcome indicators and argue for the sole therapeutic effect of TKM.

The limitations of this study are as follows. First, the trend was analyzed through CAGR at two points in 2008 and 2017, although the 10-year annual statistical data are available. However, input and output have increased linearly every year in both TKM and CM. Therefore, according to CAGR, the interpretation of the results, not based on the complex regression analysis, would not have changed significantly. The CAGR could rather have shown the trend simply and clearly. 

Second, this study explained the phenomenon and reasons based on input and output data. The results of medical use are evaluated through the measurement of outcomes. Therefore, if the results were interpreted by comparing the outcomes of CM and TKM, more convincing conclusions could have been drawn. However, few studies compare the outcomes of CM and TKM. In addition, as mentioned above, since most patients concurrently use CM and TKM, the measurement of the sole therapeutic effect of TKM is not easy. The researchers instead sought to support the argument by introducing the characteristics of the Korean health care system and the government’s TKM policies that could affect input and output.

The basis for rational decision-making on TKM policy is monitoring data. However, an in-depth research study is required to establish more specific implementation strategies. For example, it is necessary to investigate whether the status of TKM is related to lack of information accessibility, evidence or trust, public perception of TKM, or whether there are other issues. It is also necessary to steadily follow up on the impact of monitoring data-based policy decisions. Likewise, continuous monitoring is needed for the feedback process in which policy outcomes lead to another TKM policy decision.

## 5. Conclusions

In conclusion, Korea has a great statistical monitoring system for the comprehension of medical utilization. Based on this system, TKM is tasked with implementing evidence-informed policies to establish TKM services’ role and aid policymakers in making objective decisions. The current study showed that TKM accounts for a small percentage of NHS and is highly concentrated on the elderly and MRD. While the government has continually developed and operated programs that take advantage of TKM, there are still challenges in evaluating whether input and output lead to outcomes. In the case of TKM, various TM indicators monitored by Korea and policies based on them will give implications for other countries.

## Figures and Tables

**Table 1:** Changes in the number of Traditional Korean Medicine personnel and facilities in Korea (2008–2017).

	TKM Personnel			TKM Facilities		
	TKM Doctors	Doctors Who Have Both Licenses ^(a)^	HM Pharmacists ^(b)^	TKM Hospitals	TKM Clinics	HM Pharmacies
YR 2008 (n (%)) ^(c)^	14,818 (13.4)	189 (1.3)	1222 (3.7)	146 (5.8)	11,334 (22.0)	510 (2.4)
YR 2017 (n (%)) ^(c)^	24,627 (16.4)	316 (1.3)	2404 (6.1)	312 (8.7)	14,155 (22.6)	595 (2.7)
Annual average growth rate(%) ^(d)^ (2008–2017)	5.8	5.9	7.8	8.8	2.5	1.7

Abbreviations: CM, Conventional Medicine; HM, Herbal Medicine; NHI, National Health Insurance; TKM, Traditional Korean Medicine. ^(a)^ Medical doctors who got both licenses in CM and TKM. ^(b)^ HM pharmacists specialized in dispensing herbal medicines. ^(c)^ Calculation: TKM doctors (%) = No. of TKM doctors ÷ No. of (CM doctors + Dentists + TKM doctors); Doctors who have both licenses (%) = No. of doctors who have both licenses ÷ No. of TKM doctors; HM pharmacists (%) = No. of HM pharmacists ÷ No. of (CM pharmacists + HM pharmacists); TKM hospitals (%) = No. of TKM hospitals ÷ No. of (CM hospitals + Dental hospitals + TKM hospitals); TKM clinics (%) = No. of TKM clinics ÷ No. of (CM clinics + Dental clinics + TKM clinics); HM pharmacies (%) = No. of HM pharmacies ÷ No. of (CM pharmacies + HM pharmacies). Only TKM doctors and HM pharmacists are specialized in traditional medicine, and nurses are not applicable. ^(d)^ Calculated from Compound Annual Growth Rate (CAGR).

**Table 2:** Medical expenditure in Traditional Korean Medicine by the type of health insurance in 2017.

	NHI		Medical Aid	Workers’ Compensation	Automobile Insurance
	Total				
		Nursing Hospitals ^(a)^			
Medical expenditure in TKM (USD, Million) (2017)	2237	96.2	146.5	3.1	495.7
% of TKM expenditure among total medical expenditure in each type of insurance (2017)	3.6	2.1	2.3	0.6	31.8
Annual average growth rate of TKM medical expenditure (%) ^(b)^ (2008–2017)	7.2	18.8 (2010–2017)	8.2	8.4	27.8 (2014–2017)
Annual average growth rate of CM medical expenditure (%) ^(b)^ (2008–2017)	8.0	17.6 (2010–2017)	5.3	−0.5	1.5 (2014–2017)

Abbreviations: CM, Conventional Medicine; NHI, National Health Insurance; TKM, Traditional Korean Medicine; ^(a)^ Medical expenditure of nursing hospitals are included in the total medical expenditure of NHI. Data are separately presented to provide additional information. The amount of 2.1% is the share of TKM medical expenditure in total nursing hospital care expenditure; ^(b)^ Calculated from Compound Annual Growth Rate (CAGR).

## Data Availability

All data are publicly available. To acquire original data, please refer to the links in Refs. [11,12,13,14,15].

## Supplementary Materials

The following are available online at https://www.mdpi.com/article/10.3390/healthcare9101379/s1, Table S1: Top 10 herbal prescriptions in the National Health Insurance in Korea in 2017, Table S2: Comparison of the top 5 diseases for the outpatient and inpatient visits between Traditional Korean Medicine and Conventional Medicine excluding musculoskeletal-related disorders in National Health Insurance in 2017 (presented as Korean Standard Classification of Diseases 7 (KCD-7) reflecting ICD-10).
