# Supplementary material for: The Provision and Utilization of Traditional Korean Medicine in South Korea: Implications on Integration of Traditional Medicine in a Developed Country
Source: Healthcare (Basel). 2021 Oct 15;9(10):1379. doi: 10.3390/healthcare9101379 (PMC8544406; doi:10.3390/healthcare9101379)
Supplement: Supplementary file 1 [file healthcare-09-01379-s001.zip › healthcare-1387480-supplementary.pdf]

Table S1. Top 10 herbal prescriptions in the National Health Insurance in Korea in 2017.

Rank	Name of the Prescription
1	Ojeok-san (五積散)
2	Gungha-tang (芎夏湯)
3	Gumiganghwal-tang / Jiuweiqianghuo-tang (九味羌活湯)
4	Yijin-tang (二陳湯)
5	Banhasasim-tang (半夏瀉心湯)
6	Socheongryong-tang (小青龍湯)
7	Samso-eum (參蘇飮)
8	Bojungikki-tang-gami / Bu Zhong Yi Qi Tang (補中益氣湯)
9	Hyangsapyeongwi-san (香砂平胃散)
10	Pyeongwi-san (平胃散)

Source: *2017 Status of Claims for Prescribed Medicines*. Health Insurance Review & Assessment Service. 2019.

Table S2. Comparison of the top 5 diseases for the outpatient and inpatient visits between Traditional Korean Medicine and Conventional Medicine excluding musculoskeletal-related disorders in National Health Insurance in 2017 (presented as Korean Standard Classification of Diseases 7 (KCD-7) reflecting ICD-10).

Rank	Traditional Korean Medicine	Total medical services
**Top 5 Diseases (outpatient settings)**		
**1**	K30 Functional dyspepsia	J20 Acute bronchitis
**2**	J00 Acute nasopharyngitis [common cold]	K05 Gingivitis and periodontal diseases
**3**	R10 Abdominal and pelvic pain	J30 Vasomotor and allergic rhinitis
**4**	R51 Headache	J03 Acute tonsillitis
**5**	J30 Vasomotor and allergic rhinitis	J06 Acute upper respiratory infections of multiple and unspecified sites
**Top 5 Disease (inpatients settings)***		
**1**	G81 Hemiplegia	A09 Infectious gastroenteritis and colitis, unspecified
**2**	I63 Cerebral infarction	H25 Age-related cataract
**3**	F00 Dementia in Alzheimer's disease (G30.-+)	J18 Pneumonia, unspecified organism
**4**	I69 Sequelae of cerebrovascular disease	K64 Hemorrhoids and perianal venous thrombosis
**5**	G51 Facial nerve disorders	F00 Dementia in Alzheimer's disease (G30.-+)

* Liveborn infants according to place of birth (Z38) and Single spontaneous delivery (O80) is less relevant to diseases and the disease that was ranked next to the Z38 and O80 was marked. Source: *2017 National Health Insurance Statistical Yearbook*. National Health Insurance Service, Health Insurance Review & Assessment Service. 2018.
